# Use of a liquid nicotine delivery product to promote smoking cessation

**DOI:** 10.1186/1471-2458-10-155

**Published:** 2010-03-24

**Authors:** Nicholas Geimer, Carl E Olson, Deborah Baumgarten, James L Kepner, Martin C Mahoney

**Affiliations:** 1 Department of Medical Oncology, Medical College of Wisconsin, Milwaukee, WI 53226, USA; 2 Department of Radiation Oncology, Columbia St. Mary's Hospital, Milwaukee, WI 53211, USA; 3 Statistics and Evaluation Center, American Cancer Society, Atlanta, GA 30329, USA; 4 Departments of Health Behavior and Medicine, Roswell Park Cancer Institute, Buffalo, NY 14263, USA

## Abstract

**Background:**

Despite access to various pharmacotherapies and counseling support to aid cessation, smokers typically demonstrate quit rates below 50%. This report describes the results of a Phase 2a study exploring the efficacy of a liquid nicotine delivery system as an aid to smoking cessation assessed after 12 weeks of therapy.

**Methods:**

A single-arm Phase 2a study was conducted. Community-based smokers (ages 18+ years, smoking at least 10 cigarettes daily for the past year and interested in making a quit attempt) were recruited and completed clinic visits at 2 week intervals over the 12 week study period where carbon monoxide levels were assessed and the Smoke-Break product was rated on taste and overall satisfaction. Participants were provided with a supply of liquid nicotine cigarettes (e.g., Smoke-Break) at each clinic visit. A total of 69 smokers were enrolled and received the intervention product (intention to treat group, ITT) and 52 smokers verified participation (according to protocol group, ATP).

**Results:**

The cessation rate at 12 weeks after the baseline visit, assessed as the bioverified point prevalence of abstinence, was 71.1% (95% confidence interval [CI] 58.8%-83.5%) in the ATP group and 53.6% (41.8%-65.4%) in the ITT group. Participants rated the liquid nicotine delivery system highly and also expressed general satisfaction. Few adverse events were identified with no serious adverse events.

**Conclusions:**

These results support the efficacy of the liquid nicotine delivery system in smoking cessation. If this nicotine delivery product proves to be effective in larger trials, it could represent an inexpensive, readily accessible and well-tolerated agent to promote smoking cessation.

**Trial Registration:**

This trial is registered at clinicaltrials.gov as study NCT00715871.

## Background

Cigarette smoking represents the single most avoidable cause of premature death in the United States (US) and is responsible for at least 443,000 deaths annually[1]. While the prevalence of smoking in the US has declined over the past half century, the current smoking rate is about 20%, and with about 40 million adult smokers, this behavior will continue to influence rates of premature morbidity and mortality for years to come[2]. Most smokers report that they want to quit and approximately 40% attempt to stop smoking annually. Unfortunately, most quit attempts are unplanned and usually only last a few days or weeks and are unsupported by the provision of pharmacotherapy and counseling support. Difficulty quitting is best predicted by how much one smokes on a daily basis and smoking within 30 minutes of waking up each day, both of which are measures of nicotine dependence [3,4].

The Public Health Service guidelines for treating tobacco use and dependence, last updated in 2008, endorse the use of several proven pharmacotherapies for cessation including nicotine replacement (e.g., patch, lozenge, gum/resin, inhaler, and nasal spray), bupropion (Wellbutrin^®^/Zyban^®^) and varenicline (Chantix^®^), as well as the combination of counseling support and pharmacotherapy [5,6]. Quit rates for nicotine replacement therapy (NRT) range between 20-24%,[7] compared to 30% for bupropion [8] and 44% for varenicline [9,10]. While somewhat different definitions for cessation, and variable time intervals were used across these studies, complicating direct comparisons, bupropion and varenicline have greater efficacy, but require an office visit to get a prescription and are generally more costly compared to several formulations of NRT available over the counter. As a result, population efficacy for a smoking cessation product reflects the combination of product effectiveness and population reach.

This report describes the results of a Phase 2a study exploring the efficacy of a liquid nicotine delivery system as an aid to smoking cessation assessed after 12 weeks of therapy.

## Methods

### Objective

To explore the efficacy of the liquid nicotine delivery product as an aid to smoking cessation as assessed after 12 weeks of therapy. Secondary objectives included an assessment of product taste, overall satisfaction, and monitoring of adverse events.

### Design

A single arm Phase 2a study examining the efficacy of a liquid nicotine cigarette as an aid to smoking cessation.

### Participants

Eligibility criteria included smokers ages > = 18 years, 10+ cigarettes daily, smoking for at least the past year, interest in making a quit attempt, not using any other form of nicotine replacement therapy, and no known allergy to any components of the liquid nicotine cigarette (Smoke-Break). Pregnant or nursing females, persons reporting a history of heart disease or diabetes, and/or use of a prescription medication for depression or asthma were ineligible to participate. Women with child bearing potential were advised to use appropriate birth control methods during their course of treatment.

**Figure 1:**
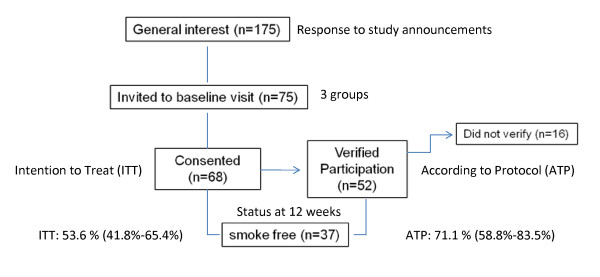
**Identification of study participants in smoking cessation trial**.

Smokers were recruited from the community via television news stories, flyers and word of mouth which generated calls from about 175 individuals. As shown in Figure 1, 75 smokers who appeared to be eligible were invited to a baseline visit.

During the baseline visit, eligibility was confirmed, study procedures were reviewed, informed consent was completed, and an initial 2 week supply of the Smoke-Break product was distributed. All participants were instructed to stop smoking by their follow-up appointment in 2 weeks. General materials on quitting smoking were available at the baseline visits. Participants were also asked to confirm their participation and their quit date by leaving a phone message within 72 hours of their baseline visit. The intention to treat (ITT) group included all participants who completed informed consent at the baseline visit and received an initial supply of Smoke-Break product (n = 69) while the according to protocol (ATP) group included all participants who confirmed participation (n = 52). This research study was reviewed and approved by the New England Institutional Review Board (#07-211).

### Intervention

Participants were provided with liquid nicotine cigarettes (e.g., Smoke-Break product) which are plastic tubes containing a 1.5 milligram (mg) dose of nicotine in a naturally flavored, artificially sweetened gel. All components are FDA-approved for use in food and pharmaceutical products.

Subjects were advised on use of Smoke-Break liquid cigarette tubes based on their daily nicotine intake estimated using the published nicotine content (in milligrams, mg) of their usual cigarette brand multiplied by the number of cigarettes smoked per day. This estimate of total daily nicotine intake was divided by 1.5 (the amount of nicotine (in mg) in each liquid cigarette tube to yield the total tubes of product to be used each day. Participants were advised not to exceed 4 tubes within a one hour period or 40 liquid cigarette tubes in a day. Subjects were provided a sufficient number of tubes to last 2 weeks. Participants returned at 2 week intervals for follow-up visits where vital signs, potential side effects, use of the liquid cigarette product and carbon monoxide levels were assessed; additional nicotine replacement product (a 2 week supply) was distributed at these visits for a total of 12 weeks of treatment. Subjects also rated the liquid cigarette product on taste and overall satisfaction, each based on a 10 point scale (1-worst, 10-best) at each follow-up visit.

### Outcomes

Consistent with established guidelines, cessation was defined as an exhaled carbon monoxide (CO) level of less than or equal to 8 parts per million (ppm) [11,12]. Participants who failed to complete appointments were assumed to have returned to smoking.

### Statistics

Sample size was determined using a one-sample chi-squared statistic with inputs being a 5% type 1 error rate, a null hypothesis that the proportion of persons able to stop smoking using nicotine replacement therapy is 0.22 [6,7], and 0.80 to be the probability of correctly concluding efficacy if the true success proportion is 0.395 (= 0.22 + 0.175). Using the design calculator NQuery 7.0, the required sample size is 50. Had the design been one-sided and based on the one-sample exact binomial distribution, the same inputs would have required a sample of at least 44 subjects to conclude an increased quit rate. Thus, the ITT and ATP sample sizes are sufficiently large to accurately assess product effectiveness. Demographic characteristics of those who confirmed and those who failed to confirm participation were compared using a chi-squared distribution. Product satisfaction and adverse effects are summarized using descriptive statistics. SPSS software (version 16.0, SPSS, Inc., Chicago. IL) was used to facilitate analyses.

## Results

Selected demographic and tobacco use characteristics of study participants are summarized in table 1. Among the 69 consented participants, 53.6% were female, 91.3% were white, 29.0% were ages 35-44 years, 40.6% were 45-54 years and the remainder were age 55 year or older. Most participants reported smoking either 20-29 daily cigarettes (44.9%) or 30+ daily cigarettes (43.5%) and 85.5% reported having smoked for at least 20 years. About 3/4 of study participants indicated that they smoked their first cigarette within 30 minutes of awakening consistent with significant nicotine addiction [3,4]. The only variable which differed among those who confirmed participation following the baseline visit and those who did not was race, with non-whites less likely to confirm participation. There were no serious adverse events and no clinically meaningful variation in the vital signs of the participants.

**Table 1:** Selected demographic and tobacco use characteristics among study participants

		confirmed participation (n = 52)		failed to confirm participation (n = 17)		
characteristic	category	n	%	n	%	p-value:
gender	female	26	50.0%	11	64.7%	p = 0.403
	male	26	50.0%	6	35.3%	
						
age group	25-44 yrs	13	25.0%	7	41.2%	p = 0.435
	45-54 yrs	22	42.3%	6	35.3%	
	55-68 yrs	17	32.7%	4	23.5%	
						
race	white	51	98.1%	12	70.6%	p = 0.003
	non-white	1	1.9%	5	29.4%	
						
# daily cigarettes	<20 cigs	6	11.5%	2	11.8%	p = 0.622
	20-29 cigs	20	42.3%	9	52.9%	
	30+ cigs	24	46.2%	6	35.3%	
						
years smoked	<20 yrs	7	13.5%	3	17.6%	p = 0.909
	20-29 yrs	25	48.1%	8	47.1%	
	30+ yrs	20	38.5%	6	35.3%	
						
pack-years smoked	<30	14	26.9%	7	41.2%	p = 0.536
	30-44	18	34.6%	5	29.4%	
	45-88	20	38.5%	5	29.4%	
						
time to 1^st ^cigarette	<30 min	37	71.2%	14	82.4%	p = 0.528
	30+ min	15	28.8%	3	17.6%	

Intention to treat (ITT) group includes all 69 participants while the according to protocol (ATP) group includes the 52 participants who confirmed participation.

The rate of smoking cessation 12 weeks after the baseline visit, measured as the bioverified point prevalence of abstinence, was 71.1% (95% confidence interval [CI] 58.8%-83.5%) in the ATP group and 53.6% (41.8%-65.4%) in the ITT group. In the ATP group, cessation rates ranged from 84.9% at week 4 to 62.3% at week 8, while in the ITT group cessation rates were 65.2% at week 4 and 47.8% at week 8.

Participants consistently rated the Smoke-Break liquid nicotine system highly on taste (median = 8 on a 10 point scale) at all follow-up visits. Likewise, participants consistently indicated robust overall satisfaction (median = 7) with the liquid nicotine cigarette product. Rating did not differ among those who achieved cessation and those who did not.

Overall, 17 of 52 participants (32.7%) who returned for follow-up assessments reported some adverse event; no one reported more than one potential adverse event. As shown in table 2, only one of these was felt to be probably attributable to the liquid cigarette nicotine delivery product. In all cases, reported symptoms fully resolved. No serious adverse events occurred.

**Table 2:** Reports of adverse events among study participants (n = 52), with severity and attribution

adverse event	# reporting	severity	attribution
diarrhea/loose stools	3	mild	not attributable (n = 1); possible (n = 2)
dizzy/light headed	2	mild	not attributable (n = 1); possible (n = 1)
stomach pains/gas	1	mild	possible
heartburn	1	mild	not attributable
gum swelling	1	mild	not attributable
mouth sores	1	mild	not attributable
sore throat	1	mild	not attributable
nasal dryness/irritation	1	mild	not attributable
upper respiratory congestion	1	mild	not attributable
finger dislocation	1	mild	not attributable
sleep problems	1	mild	possible
skin infection	1	mild	not attributable
eye infection	1	mild	not attributable
constipation	1	mild	not attributable

## Discussion

Results from this single-arm Phase 2a study support the efficacy of the liquid nicotine cigarette as an aid to achieving smoking cessation. These estimates of cessation, bioverified through measurement of exhaled CO, are interesting and warrant confirmation in subsequent Phase 3 studies. It is possible that the manipulation of the liquid cigarette product during use serves to provide important tactile and oral stimulation to smokers during their quit attempt.

Abstinence may be defined in a variety of ways[12]. Similar to approaches utilized for exploring new therapeutic interventions, this proof of concept trial relied upon a measure of cessation at limited time points (e.g., point prevalence assessments at 2 week follow-up visits over an interval of 12 weeks). It is important to note that this measure of abstinence provides a useful and relevant estimate of treatment effect size. In other words, these results support the efficacy of this alternative nicotine replacement delivery formulation for smoking cessation. However, until randomized comparative trials are completed, we are unable to conclude whether the efficacy of the Smoke-Break product exceeds that observed with other forms of NRT including patches, gum/resin and lozenges.

Continuous abstinence likely correlates strongly with long term abstinence[12]. Data from this study suggest a high rate of continuous abstinence, defined as abstinence between 2 follow-up visits, among a majority of our study participants. For example, 29 of the 37 (78%) participants who were smoke-free at the week 12 visit appeared to be continuously abstinent between visits at week 4 and week 12. However, this interpretation is based on repeated measures of point prevalence abstinence as assessed at 2 week intervals, which likely overestimates the true rate of continuous abstinence since the detection window for CO is approximately 24 hours[11]. On the other hand, it would be difficult for participants to be smoke-free only on the days of follow-up visits while continuing to smoke on other days. Allowing a 2 week or 4 week grace period to assess continuous abstinence allows subjects to gain experience with the therapeutic intervention and also to experience slips and relapses that often occur in many smokers trying to quit[12]. In addition, point prevalence measures of abstinence also acknowledge the slips and relapses which occur during a quit attempt. Potential biases and limitations of this Phase 2a study include possible selection bias resulting from recruitment procedures and the likelihood that long-term quit rates, at both 6 months and 1 year, will be lower than those observed at 12 weeks.

Unlike over-the-counter (OTC) nicotine-replacement products (patch, gum, lozenge), the Smoke-Break nicotine delivery system appears to address both the "oral fixation," and "hand-to-mouth" conditioned sensory and motor aspects which characterize the smoking experience. The Smoke-Break cylinder-shaped nicotine delivery system was created to deliver a nicotine-containing, liquid formulation. When placed between the lips, the liquid in the cylinder is drawn into the mouth, much like sipping through a straw. The liquid is held in the mouth for a brief time and then swallowed partially mimicking the action of smoking.

Similar to the vast experience with over-the-counter nicotine replacement products [13], the nicotine delivery system used in this study appears to be safe and well-tolerated. Side effects of NRT mainly include local irritation (i.e., mouth sores, skin rash, nasal and throat irritation) associated with the route of administration of the medication (i.e., mouth, skin, nares); these side effects were typically mild and transient[14].

While numerous studies have shown improved efficacy when NRT is combined with professional counseling,[6] no formal counseling was made available to subjects during this study. Participants in this study were able to direct questions to the study staff about the product, and to discuss their experiences, every two weeks, in lieu of professional counseling. Subsequent clinical trials should consider a counseling component in addition to the nicotine delivery system[6].

The provision of counseling support in a subsequent trial may serve to further increase cessation rates with the liquid nicotine dispenser, although a unique strength of the present study is that the results appear to be generalizable to groups of smokers who opt not to make use of counseling services. Although compelling, these results require validation in a larger clinical trial with attention to cessation rates across a longer follow-up interval and exploration of effects among different groups of smokers.

## Conclusions

These results support the efficacy of the liquid cigarette nicotine delivery system in smoking cessation as assessed after 12 weeks of therapy. If this nicotine delivery product proves to be effective in larger trials, it could represent an inexpensive, readily accessible and well-tolerated agent to promote tobacco cessation.

## Competing interests

CEO holds an equity interest in the Smoke-Break product. MCM is a member of the Speakers Bureau at Pfizer Inc., manufacturer of Chantix^®^(varenicline). All other authors declare that they have no competing interests.

## Authors' contributions

Study conception and design: NG, CEO, acquisition of data: NG, CEO, DB, analysis and interpretation of data: JLK, MCM, drafting the article and/or revising it critically for important intellectual content: NG, CEO, DB, JLK, MCM. All authors have read and approved the final manuscript.

## Pre-publication history

The pre-publication history for this paper can be accessed here:

http://www.biomedcentral.com/1471-2458/10/155/prepub
